# Epidemiology and demographics of juvenile idiopathic arthritis in Africa and Middle East

**DOI:** 10.1186/s12969-021-00650-x

**Published:** 2021-12-02

**Authors:** Sulaiman M. Al-Mayouf, Muna Al Mutairi, Kenza Bouayed, Sara Habjoka, Djohra Hadef, Hala M. Lotfy, Cristiaan Scott, Elsadeg M. Sharif, Nouran Tahoun

**Affiliations:** 1 Pediatric Rheumatology, King Faisal Specialist Hospital and Research Center College of Medicine, Alfaisal University, Po Box 3354, Riyadh, 11211 Saudi Arabia; 2 Al Adan Hospital, Hadiya, State of Kuwait; 3 Department of Rheumatology and Pediatric Internal Medicine, University Hospital IBN Rochd, Casablanca, Morocco; 4 Pfizer Biopharmaceutical Group, Emerging Markets, Dubai, United Arab Emirates; 5 Department of Pediatrics, University Hospital Center of Batna Faculty of Medicine, Batna 2 University, Batna, Algeria; 6 Professor of Pediatrics and Pediatric Rheumatology, Cairo University, Giza, Egypt; 7 Division of Paediatric Rheumatology, Department of Paediatrics and Child Health, University of Cape Town, Cape Town, South Africa; 8 Consultant Rheumatologist, Al Jalila Children’s Specialty Hospital, Dubai, United Arab Emirates; 9 Pfizer Biopharmaceutical Group, Emerging Markets, Cairo, Egypt

## Abstract

Juvenile Idiopathic Arthritis (JIA) is a group of chronic heterogenous disorders that manifests as joint inflammation in patients aged <16 years. Globally, approximately 3 million children and young adults are suffering from JIA with prevalence rates consistently higher in girls. The region of Africa and Middle East constitute a diverse group of ethnicities, socioeconomic conditions, and climates which influence the prevalence of JIA. There are only a few studies published on epidemiology of JIA in the region. There is an evident paucity of adequate and latest data from the region. This review summarizes the available data on the prevalence of JIA and its subtypes in Africa and Middle East and discusses unmet needs for patients in this region. A total of 8 journal publications were identified concerning epidemiology and 42 articles describing JIA subtypes from Africa and Middle East were included. The prevalence of JIA in Africa and Middle East was observed to be towards the lower range of the global estimate. We observed that the most prevalent subtype in the region was oligoarticular arthritis. The incidence of uveitis and anti-nuclear antibody (ANA) positivity were found to be lower as compared to the incidence from other regions. There is a huge unmet medical need in the region for reliable epidemiological data, disease awareness, having regional and local treatment guidelines and timely diagnosis. Paucity of the pediatric rheumatologists and economic disparities also contribute to the challenges regarding the management of JIA.

## Background

Juvenile Idiopathic Arthritis (JIA) is the most common chronic heterogenous rheumatological disorder that manifests in patients aged less than 16 years and, in some cases, can cause severe impairment and disability. It constitutes various subtypes with different clinical manifestations, genetic markers, and pathogenesis [1]. According to the most commonly used classification proposed by the International League of Associations for Rheumatology (ILAR), seven different subtypes are recognized to classify patients: oligoarticular, rheumatoid factor (RF) positive polyarticular, RF negative polyarticular, enthesitis related arthritis (ERA), systemic onset, psoriatic arthritis, and undifferentiated arthritis [1, 2].

The precise cause and pathogenesis of JIA are unknown; however, genetic, environmental, and autoimmune factors are hypothesized to play a role in the development of JIA [3, 4]. Socioeconomic status is associated with delayed access to rheumatology care and worsening disease severity in JIA patients, directly affecting their well-being and quality of life [5].

Globally, approximately 3 million children and young adults are estimated to suffer from JIA [6, 7]. The global prevalence of JIA has been estimated to range from 3.8 to 400/100,000 with an incidence of 1.6 to 23/100,000 [8]. Girls were consistently found to be at a higher risk than boys, and oligoarticular subtype was found to be predominant [8].

Africa and Middle East countries constitute a diverse group of ethnicities, socioeconomic backgrounds, and climatic conditions. Few studies have assessed the prevalence of JIA in the region and there is a paucity of adequate and latest data from the region on the epidemiology of JIA. A comprehensive understanding of JIA in the regions is required.

Given the social, economic, and cultural diversity of African and Middle Eastern countries, many studies conducted in this region may underestimate the prevalence of JIA. The aim of this review article was to critically assess and summarize the available published data on epidemiology and demographics of JIA in the Africa and Middle East region and highlight the unmet needs of the region and current efforts being undertaken in the region to generate quality data on JIA and the way forward to address the lacunae. The unmet needs section describes unique challenges from the region by the authors from independent references.

## Methods

Our methodology for searching the NCBI PubMed database included the following search strings: “((juvenile idiopathic arthritis) OR JIA) AND (Africa OR (Middle East) OR AfME) AND prevalence.” Search terms also included “Juvenile Chronic Arthritis” and “Juvenile Rheumatoid Arthritis.” Additional searches were conducted to include “(Africa OR (Middle East) OR AfME)” with individual countries in the region.

Publications were included if they evaluated JIA disease prevalence in the individual African or Middle Eastern countries or in African and Middle Eastern regions, using prospective or retrospective study designs or a systematic review or meta-analysis approach between May 1988 to April 2021. We included both population based and hospital-based studies. Prevalence rates were extracted from the articles and were not estimated.

For demographic section, publications were included if they evaluated JIA disease subtype and characteristics in individual African of Middle eastern countries or region between May 1988 to April 2021.

From the articles summarizing epidemiology data from the region, parameters extracted were region/country, prevalence, incidence, sample size, number of cases, classification criteria, age range, study period, and study design (population and setting) were included in (Table 1).

**Table 1:** Epidemiology of Juvenile Idiopathic Arthritis in Africa and Middle East

Sr. No.	Reference	Region/ Country	Prevalence	Incidence	No. of cases	***N***	Classification Criteria	Age Range	Study Years
**Region**									
1	Usenbo et al., 2015 [10]	Africa	(0.1-3.43)/100,000(NA)	NA	NA	NA	Multiple classification criteria	0-16	1975-2014
**Country**									
2	Khuffash et al., 1988 [13]	Kuwait	22/100,000(NA)	NA	41	186,363	ACR (for 3 months)	0-11	1978-1987
3	Khuffash et al., 1990 [14]	Kuwait	18.7/100,000 (15.3-22.6)	2.8 (2.3-3.4)/100,000	108 JCA	577,540	ACR (for 3 months)	0-11	1981-1988
4	Abdwani et al., 2015 [11]	Oman	**Boys** 12/100,000 (NA)	2/100,000	107 JIA	528,480	ILAR 2004	0-13	2004-2013
			**Girls** 28/100,000 (NA)						
			20/100,000 (NA)						
5	Ozen et al., 1998 [15]	Turkey	64/100,000 (43-91)	NA	30 JCA	46,813	EULAR (for 6 weeks)	0-15	1997
6	El-Soud et al., 2013 [12]	Egypt Sharkia Governate, Egypt	3.43/100,000 (3.1–4.3)	NA	132 JIA	3,844,718	2004 ILAR	0-15	2009-2010
			boys 2.58/100,000 (2.4–3.6)						
			Girls 4.33/100,000 (3.3–5.1)						
7	Singwe-Ngandeu et al., 2013 [16]	Cameroon	1/100,000 (0.7-1.3)	NA	35	34,782	Not reported	NA	2004-2012
8	Tayel et al., 1999 [17]	Egypt Alexandria	3.3/100,000 (4-62)	NA	NA	1500	EULAR	10-15years	NA

ACR, American College of Rheumatology Association; EULAR, The European League Against Rheumatism; ILAR, International League of Associations for Rheumatology; JCA, juvenile chronic arthritis; JIA, juvenile idiopathic arthritis; NA, not applicable.

From the articles summarizing demographic data from the region, parameters such as country, number of cases, female to male ratio, mean age of onset (years), distribution of subtypes, presence, definition and methodology of testing for antinuclear antibody (ANA) positivity, uveitis, Rheumatoid factor (RF) positivity, and human leukocyte antigen HLA-B27 were extracted and included in (Table 2).

**Table 2:** Demographic Characteristics

Sr. No.	Reference	Country	N (no. of cases)	F:M	Mean Age of onset (years)	Subtype			ANA positivity		Uveitis		RF positivity		HLA-B27	
						Type	No.	%	%	Methodology of testing	%	Methodology/ Nature	%	Methodology of testing	%	Methodology of testing
**Regional**																
1	Consolaro et al., 2019 [22]	Africa and Middle East	1209	1.61	6·0 (2·9–9·8)*	Psoriatic arthritis	37	3.1	NA	NA	5.9	NM	NA	NA	NA	NA
						RF-positive polyarthritis	61	5.0								
						Undifferentiated arthritis	68	5.6								
						ERA	111	9.2								
						Systemic	204	16.9								
						RF-negative polyarthritis	271	22.4								
						Oligoarticular	457	37.8								
2	Al-Mayouf et al., 2021 [28]	Arab (Saudi Arabia, Libya, United Arab Emirates, Jordan, Oman, Egypt, Kuwait)	702	2.04	5 (IQR 2.0- 9.0)*	Undifferentiated	11	1.6	30.9	Immunoassay	8.3	NM	9.3	Immunoassay RF was tested at least twice, with a minimum of 3 months apart. Test results were interpreted according to cutoff values of the local laboratories.	5.3^#^	Flow cytometry
						Psoriatic	28	3.9								
						ERA	39	5.6								
						Oligoarticular Extended	43	6.1								
						Polyarticular RF positive	48	6.8								
						Polyarticular RF negative	159	22.6								
						Systemic	172	24.5								
						Oligoarticular persistent	202	28.8								
**Country**																
3	Khuffash et al., 1988 [13]	Kuwait	41	1.28	NA	Oligoarticular ANA negative	4	9.8	NA	NM	NA	NA	NA	NA	NA	NA
						Polyarticular seropositivity	5	12.2			NA					
						Oligoarticular ANA positive	5	12.2	12.2		60.0^†^	Asymptomatic chronic uveitis detected by slit lamp examination by an ophthalmologist				
						Systemic polyarticular	6	14.6	NA		NA	NA				
						Systemic oligoarticular	10	24.4			NA					
						Polyarticular seronegative	11	26.8			NA					
4	Khuffash et al., 1990 [14]	Kuwait	108	1.04	NA	Oligoarticular seropositive	3	2.8	NA	NA	NA	NA	NA	NA	NA	NA
						Oligoarticular ANA positive	9	8.3	8	NM						
						Polyarticular seropositive	10	9.3	NA	NA						
						Systemic polyarticular	13	12.0								
						Systemic oligoarticular	18	16.7								
						Oligoarticular ANA negative	19	17.6								
						Polyarticular seronegative	36	33.3								
5	Abdwani et al., 2015 [11]	Oman	107	2.57	6.85 ± 3.86 years	Psoriatic	1	0.9	32	Indirect Immunoflorescence; titer of ≥1:80 obtained on at least 1 clinic visit during the disease course was considered positive	None	Slit lamp examination by ophthalmologist during regular follow up visits at 3, 6 or 12 monthly intervals as per the pediatric screening recommendation	7.5	ELISA; RF was considered to be positive when titers were >20 IU/ml (If only one test of RF was performed, which was the case for many patients, then the results of this test were used to assign a JIA subtype rather than apply the subtype category of “other JIA.”)	NA	NA
						ERA	3	2.8								
						Polyarticular RF positive	8	7.5								
						Systemic JIA	19	17.8								
						Oligoarticular JIA	34	31.8								
						Polyarticular RF negative	42	39.3								
6	Ozen et al., 1998 [15]	Turkey	30	0.67	NA	Systemic	1	12.5	NA	NA	NA	NA	NA	NA	NA	NA
						Polyarticular	12	42.4								
						Oligoarticular	17	45.1								
7	Abou El-Soud et al., 2013 [12]	Egypt	132	1.59	12.5 ± 4.56	Systemic	18	13.6	48.5	Indirect immunofluorescence on Hep-2 cells, with positive titers from 1/40 with at least two determinations 3 months apart during the first 6 months of the disease	19.7	Detected by slit lamp examination	27.20	Semi-quantitative latex test; titers ≥30 IU/mL were considered positive with at least two determinations 3 months apart during the first 6 months of the disease	66^‡^	Low-resolution PCR analysis
						ERA	6	4.5								
						Polyarticular RF positive	11	8.3								
						Polyarticular RF negative	28	21.2								
						Oligoarticular	69	52.3								
8	Furia et al., 2020 [47]	Tanzania	28	1.15	NA	Oligoarticular	1	3.6	0.0	NM	NA	NA	NA	NA	NA	NA
						Systemic	6	21.4								
						Polyarticular	21	75.0								
9	Aiche et al., 2018 [31]	Algeria	70	1.8	7.3*	Psoriatic	1	1.4	2.9	NM	1.5	NM	NA	NA	NA	NA
						Systemic	7	10.0								
						ERA	8	11.4								
						Polyarticular RF positive	14	20.0								
						Polyarticular RF negative	15	21.4								
						Oligoarticular	25	35.7								
10	Al Marri et al., 2017 [32]	Saudi Arabia	23	6.67	3.5	Psoriatic	1	4.3	8.7	NM	NA	NA	13.0	NM	NA	NA
						Polyarticular RF positive	3	13.0								
						Polyarticular RF negative	5	21.7								
						Systemic	14	60.9								
11	Al-Mayouf et al., 2018 [35]	Saudi Arabia	100	1.70	4.5*	ERA	3	3.0	15.0%	NM	8.1%	NM	NA	NA	NA	NA
						Undifferentiated	3	3.0								
						Psoriatic	6	6.0								
						Polyarticular RF positive	13	13.0								
						Oligoarticular	23	23.0								
						Polyarticular RF negative	25	25.0								
						Systemic	27	27.0								
12	Salah et al., 2009 [63]	Egypt	196	1.09	6.257±3.41 years	Systemic-onset	47	24.0	21.7	Indirect immunofluorescence; positive at serum dilution between 1:80 to 1:60	5.6	Slit lamp examination; all detected patients had chronic uveitis	NA	NA	NA	NA
						Polyarthriticular	68	34.7								
						Extended oligoarticular	18	9.2								
						Persistent Oligoarticular	63	32.1								
						Oligoarticular	81									
13	Al-Abrawi et al., 2018 [33]	Oman	57	2.35	5.9*	ERA	0	0.0	7.0	NM	0	NM	NA	NA	NA	NA
						Undifferentiated	0	0.0								
						Psoriatic	2	3.5								
						Polyarticular RF positive	6	10.5								
						Systemic	13	22.8								
						Oligoarticular	16	28.1								
						Polyarticular RF negative	20	35.1								
14	Demirkaya et al., 2018 [45]	Turkey	466	1.49	6.3 (2.7–10.8)	Polyarticular RF positive	11	2.4	9.9	NM	8.1	NM	NA	NA	NA	NA
						Undifferentiated	12	2.6								
						Psoriatic	15	3.2								
						Systemic	64	13.7								
						ERA	70	15.0								
						Polyarticular RF negative	105	22.5								
						Oligoarticular	189	40.6								
15	El Miedany et al., 2018 [46]	Egypt	100	0.89	9.2 (5.3–11)*	Polyarticular RF positive	2	2.0	0.0	NM	6.0	NM	NA	NA	NA	NA
						Psoriatic	2	2.0								
						ERA	2	2.0								
						Oligoarticular	10	10.0								
						Systemic	20	20.0								
						Polyarticular RF negative	24	24.0								
						Undifferentiated	40	40.0								
16	Hashad et al., 2018 [48]	Libya	100	2.33	6.4 (3.1-10.4)*	Psoriatic	4	4.0	7.0	NM	2.0	NM	NA	NA	NA	NA
						Polyarticular RF positive	5	5.0								
						Undifferentiated	5	5.0								
						ERA	13	13.0								
						Systemic	22	22.0								
						Polyarticular RF negative	25	25.0								
						Oligoarticular	26	26.0								
17	Oyoo et al., 2016 [55]	Kenya	68	2.4	8.45	ERA	4	5.9	10.9^§^	NM	1.47	Slit lamp examination by an ophthalmologist	17.6^¶^	One positive or negative RF assay was considered adequate to classify polyarticular patients	NA	NA
						Systemic JIA	10	14.7								
						Polyarticular RF positive	12	17.6								
						Oligoarticular arthritis	16	23.5								
						Polyarticular RF negative	26	38.2								
18	Scott et al., 2018 [57]	South Africa	91	1.68	5.9*	Systemic	4	4.4	2.2	NM	8.2	NM	NA	NA	NA	NA
						Polyarticular RF positive	6	6.6								
						Psoriatic	6	6.6								
						Undifferentiated	8	8.8								
						ERA	14	15.4								
						Polyarticular RF negative	21	23.1								
						Oligoarticular	32	35.2								
19	Sen et al., 2015 [58]	Turkey	213	1.07	8.1 (range 8 months-15.4 years)	Psoriatic	2	0.90	11.70	Immunofluorescent antibody method; titers >160 IU/mL were considered positive	4.20	Slit lamp examination by an ophthalmologist	13.10	Nephelometric method; positivity defined by titers >20 U/mL on at least two occasions during the first six months of disease onset	2.8^	PCR
						Undifferentiated	0	0.00								
						Systemic	19	8.90								
						Polyarticular RF positive	23	10.80								
						ERA	23	10.80								
						Polyarticular RF negative	67	31.50								
						Oligoarticular	79	37.10								
20	Shafaie et al., 2018 [59]	Iran	102	2.19	5.2*	ERA	0	0.0	2.9	NM	1.0	NM	NA	NA	NA	NA
						Undifferentiated	0	0.0								
						Polyarticular RF positive	1	1.0								
						Psoriatic	1	1.0								
						Systemic	15	14.7								
						Polyarticular RF negative	16	15.7								
						Oligoarticular	69	67.6								
21	Yener et al., 2020 [61]	Turkey	116	1.58	NA	Undifferentiated	0	0.0	44**	Immunofluorescence; titer of 1/100 was considered positive	2.6	Slit lamp examination by an ophthalmologist every 6 months	22.7^##^	Two RF values above 10 U/L measured at an interval of 3 months in a 6-month period were considered significant	21.1^††^	Positive or negative for antigen
						Psoriatic	4	3.4								
						Systemic	15	12.9								
						Polyarticular RF positive	5	4.3								
						Polyarticular RF negative	17	14.7								
						Oligoarticular	37	31.9								
						ERA	38	32.8								
22	Çakan et al., 2017 [41]	Turkey	265	0.95	NA	Undifferentiated	5	1.9	27.20	Indirect Immunofluorescence; titers ≥1:100 were classified as positive	4.5	All cases were of anterior uveitis	3.8	Verified by a second analysis at least 3 months later	26^‡‡^	NM
						Psoriatic JIA	5	1.9								
						Polyarticular RF positive	10	3.8								
						Systemic JIA	35	13.2								
						Persistent oligoarticular	81	30.6								
						Polyarticular RF negative	36	13.5								
						Extended Oligoarticular JIA	6	2.3								
						ERA	87	32.9								
23	Kasapçopur et al., 2004 [51]	Turkey	198	0.87	6.62 ± 4.12	Other	5	2.5	18.2	Hep-2 cell; titers above 1/40 were considered positive	10.1	Slit lamp and a detailed ophthalmologic examination by ophthalmologist; single evaluation was considered sufficient for uveitis positivity; repeated every 3 months in uveitis and ANA positive patients	3.5	Nephlometric method	NM	Histocompatibility antigen determination
						Polyarticular RF positive	7	3.5								
						Extended Oligoarticular	9	4.5								
						Psoriatic JIA	11	5.6								
						Polyarticular RF negative	34	17.2								
						Oligoarticular JIA	37	18.7								
						ERA	43	21.7								
						Systemic JIA	52	26.3								
24	Ozdogan et al., 1991 [56]	Turkey	147	0.77	8.4±3.9	Juvenile spondylitis	3	2.0	5.6	Indirect Immunofluorescence using human leukocytes as nuclear substrate and fluorescein anti IgG antisera	7.5	Slit lamp examination; Chronic uveitis in 7 patients and acute anterior uveitis in 1 male patient	10	Latex slide agglutination test	45	Standard microcytotoxicity test
						Polyarticular sero-positive	7	5.0								
						Polyarticular sero-negative	19	13.0								
						Systemic	37	25.0								
						Pauciarticular	81	55.0								
25	Abdul-Sattar et al., 2014 [30]	Egypt	52	2.06	NA	Polyarticular RF positive	5	10.0	NA	NA	NA	NA	NA	NA	NA	NA
						Oligoarticular persistent	9	17.0								
						Polyarticular RF negative	11	21.0								
						Systemic	12	23.0								
						Oligoarticular extended	15	29.0								
26	Abdul-Sattar et al., 2014 [29]	Egypt	58	2.41	NA	Polyarticular RF positive	5	8.6	NA	NA	NA	NA	NA	NA	NA	NA
						Oligoarticular persistent	11	19.0								
						Polyarticular RF negative	12	20.7								
						Systemic	13	22.4								
						Oligoarticular extended	17	29.3								
27	Albokhari et al., 2019 [36]	Saudi Arabia	44	1.59	NA	ERA	0	0.0	NA	NA	NA	NA	NA	NA	NA	NA
						Psoriatic	2	4.5								
						Oligoarticular	6	13.6								
						Polyarticular	7	15.9								
						Systemic	12	27.3								
						Unknown	17	38.6								
28	Al-Hemairi et al., 2015 [34]	Saudi Arabia	82	1.65	7.1 ± 3.6 year	Undifferentiated	0	0.0	36.58	ELISA; titer of 1:80 or more was considered positive. Positivity was confirmed only if two samples were positive at least three months apart	8.53	Slit lamp examination by an ophthalmologist	4.87^§§^	RF positivity was confirmed only if two samples were positive, tested three months apart	100% in ERA	NM
						ERA	1	1.2								
						Polyarticular RF positive	4	4.9								
						Psoriatic	4	4.9								
						Polyarticular RF negative	20	24.4								
						Oligoarticular	23	28.0								
						Systemic	30	36.6								
29	Amine et al., 2009 [38]	Morocco	80	1.42	7.53	Extended oligoarticular	4	5.0	NA	NA	NA	NA	NA	NA	NA	NA
						Systemic	21	26.0								
						Polyarticular	25	31.5								
						Persistent oligoarticular	30	37.5								
30	Bahabari et al., 1997 [39]	Saudi Arabia	115	1.21	6(0.75-16)	ERA	0	0.0	30.0	Indirect immunofluorescence; positive at serum dilution between 1:80 to 1:60	1.70	Chronic uveitis	10.0	Slide agglutination test (till 1991); ELISA (after 1992)	6.0 ^¶¶^	Standard microcytotoxicity
						Polyarticular RF positive	12	10.4								
						Polyarticular RF negative	23	20.0								
						Oligoarticular	30	26.1								
						Systemic	50	43.5								
31	Bouaddi et al., 2013 [40]	Morocco	33	0.83	NA	Polyarticular RF negative	1	3.0	76	NM	NA	NA	12.10	NM	NA	NA
						Oligoarticular	4	12.1								
						ERA	5	15.2								
						Systemic	8	24.2								
						Polyarticular RF positive	15	45.5								
32	Chipeta et al., 2013 [42]	Zambia	78	1.23	8.70 years (range: 1–15 years)	Psoriatic	1	1.3	NA	NA	11.50	Chronic uveitis in 3 patients with oligoarticular JIA and in 2 patients with ERA; Acute uveitis in 1 each of ERA and polyarticular JIA	NA	NA	NA	NA
						ERA	5	6.4								
						Polyarticular RF positive	9	11.5								
						Systemic	11	14.1								
						Oligoarticular	25	32.1								
						Polyarticular RF negative	27	34.6								
33	Hussein et al., 2018 [49]	Egypt	63	0.90	6.1 (range 3-14) ±2.8	ERA	0	0.0	20.6	NM	6.3	Slit lamp examination	69.8	NM	NA	NA
						Undifferentiated	0	0.0								
						Psoriatic Arthritis	0	0.0								
						Polyarticular RF negative	6	9.5								
						Systemic	15	23.8								
						Polyarticular RF positive	16	25.4								
						Oligoarticular	26	41.3								
34	Olaosebikan et al., 2017 [54]	Nigeria	28	NA	NA	Systemic	5	17.9	NA	NA	NA	NA	7.14^^	Nephlometry	NA	NA
						Oligoarticular	9	32.1								
						Polyarticular	14	50.0								
35	Weakley et al., 2012 [60]	South Africa	78	1	8 (4–10)*	Psoriatic Arthritis	1	1.3	3.8***	Majority ELISA, remaining Hep 2 immunofluorescent	NA	NA	14.1^^^	One positive or negative assay for RF was considered sufficient to classify a patient with polyarthritis	23^###^	NM
						Oligoarticular Extended	4	5.1								
						Systemic	6	7.7								
						Polyarticular RF positive	11	14.1								
						Persistent Oligoarticular	17	21.9								
						ERA	18	23.0								
						Polyarticular RF negative	21	26.9								
36	Mostafa et al., 2019 [53]	Egypt	48	2.42	NA	Psoriatic	0	0.0	NA	NA	NA	NA	42.0	NM	NA	NA
						ERA	0	0.0								
						Oligoarticular	8	17.0								
						Polyarthritis	28	58.0								
						Systemic	12	25.0								
37	Dagher et al., 2014 [43]	Lebanon	66	1	5.2 years (range: 9 months - 14 years).	Polyarticular RF positive	0	0.0	23	NM	6.1	NM	NA	NA	NA	NA
						Oligoarticular extended	3	4.0								
						Undifferentiated	3	5.0								
						ERA	11	17.0								
						Systemic	15	23.0								
						Polyarticular RF negative	16	24.0								
						Oligoarticular persistent	18	27.0								
38	Khawaja et al., 2017 [52]	UAE	66	2.47	NA	ERA	1	1.5	NA	NA	7.6	NM	NA	NA	NA	NA
						Psoriatic	1	1.5								
						Oligoarticular extended	3	4.5								
						Polyarticular RF positive	12	18.2								
						Systemic	13	19.7								
						Oligoarticular persistent	16	24.2								
						Polyarticular RF negative	20	30.3								
39	Alzyoud et al., 2020 [37]	Jordan	210	1.23	5.08±3.4 (7 months to 14 years)	Polyarticular RF positive	8	3.8	33.60	Indirect immunofluorescence using Hep-2 cells; titers > 1/80 were considered positive	14.2^†††^	Slit lamp examination at a dedicated uveitis clinic	3.80	Nephelometry; Considered positive when titers were ≥ 15 units/mL. and at least two positive results, 3 months apart, in the first 6 months of observation	NA	NA
						ERA	15	7.1								
						Polyarticular RF negative	18	8.5								
						Psoriatic arthritis	18	8.5								
						Systemic arthritis	36	17.1								
						Persistent Oligoarticular	96									
						Extended Oligoarticular	19									
						Oligoarticular	115	54.7								
40	Demirkaya et al., 2011 [44]	Turkey	634	1.26	7.69±4.41 (1-11 years)	Psoriatic	13	2.1	30.1	Titer of 1:80 was chosen as a cut-off point for ANA positivity for at least two positive results at least 3 months apart	11.6	Defined in accordance with the criteria of the SUN Working Group^^^^	3.1^‡‡‡^	NM	63.3^§§§^	NM
						RF positive polyarthritis	20	3.2								
						Extended Oligoarticular	26	4.1								
						Systemic	92	14.5								
						ERA	120	18.9								
						RF negative polyarthritis	129	20.3								
						Persistent Oligoarticular	234	36.9								
41	Karadag et al., 2020 [50]	Turkey	281	NA	NA	RF positive polyarticular	4	1.4	NA	NA	NA	NA	NA	NA	NA	NA
						Undifferentiated	7	2.5								
						Systemic	11	3.9								
						Psoriatic	13	4.6								
						RF negative polyarticular	19	6.8								
						ERA	97	34.5								
						Oligoarticular	130	46.3								
42	Yilmaz et al., 2008 [62]	Turkey	196	0.92	6.9 ± 3.7	Psoriatic arthritis	2	1.0	14.2	Indirect Immunofluorescence using Hep-2 cell; titers >1/80 were considered positive	2	Slit lamp and detailed ophthalmological examination by ophthalmologist every 4 – 6 months; chronic uveitis occurred in 2 patients with persistent oligoarticular JIA	8.1	Nephelometry; Considered positive when titers were 15 units/mL and confirmed with two positive results, 3 months apart, during the first 6 months of observation	5.6	Lymphocytotoxicity assay
						Others	5	2.5								
						RF (+) polyarticular JIA	13	6.6								
						Oligoarticular Extended	19	9.6								
						ERA	19	10.3								
						Systemic JIA	30	15.0								
						Oligoarticular Persistent	48	24.4								
						RF (–) polyarticular JIA	60	30.6								

* Represents values in median

^#^All patients underwent HLA-B27 testing; number patients tested not available in the article

^†^3 out of 5 oligoarticular JIA patients tested positive for uveitis, however no full cohort uveitis rate is mentioned

^‡^ HLA testing was carried out in only ERA (6 cases)

^§^Not all cases were tested (5/46; 2 oligoarticular, 3 polyarticular RF Positive)

^¶^Only in RF positive patients

^Note that HLA-B27 test was done in only 47 of the 213 JIA patients

**overall (62.22% in oligo)

^##^In polyarticular JIA

^††^in ERA

^‡‡^HLA-B27 was studied in 169 patients (in all patients with ERA phenotype and male patients over the 6 years of age)

^§§^Note that only the RF positive polyarticular patients tested positive (n=82)

^¶¶^HLA-B27 was tested in 32 patients

^^Positive in 2 polyarticular positive RF and this is not specific to JIA patients only

***ANA testing was performed only on oligoathritis patients(n=67)

^^^Performed for polyarticular subtype

^###^HLA tests were only performed for ERA subtype; all patients tested positive

^†††^Most of them were Oligoarticular JIA 25/115 (21.7%) and were associated with positive ANA in 16/115 (14%)

^‡‡‡^Tested in all except systemic; positive in only RF positive cases

^§§§^Tested only in ERA patients

^¶¶¶^In patients with uveitis; 24.6% in patients without uveitis; 28.4% combined population

^^^^Both the publications have cited *Jabs et al., 2005* for the SUN Working Group Anatomic Classification of Uveitis. SUN working group has classified uveitis based on the primary site of inflammation: anterior uveitis (anterior chamber); intermediate uveitis (vitreous); posterior uveitis (retina or choroid); panuveitis (anterior chamber, vitreous, and retina or choroid).

ANA, anti-nuclear antibody; ARA, American Rheumatology Association; ELISA, Enzyme-Linked Immunoassay; ERA, enthesitis-related arthritis; EULAR, The European League Against Rheumatism; HLA, human leukocyte antigen; ILAR, International League of Associations for Rheumatology; IQR, interquartile range; JIA, juvenile idiopathic arthritis; NA, not available (study did not assess the parameter); NM, not mentioned; PCR, polymerase chain reaction; RF, rheumatoid factor.

Additionally, online databases of the American College of Rheumatology, the Asia-Pacific League of Associations for Rheumatology and the European League Against Rheumatism, Arab League of Associations of Rheumatologists, African league of Associations of Rheumatologists, and South African Rheumatism and Arthritis Association were searched for abstracts presented at annual congresses.

**Figure 1:**
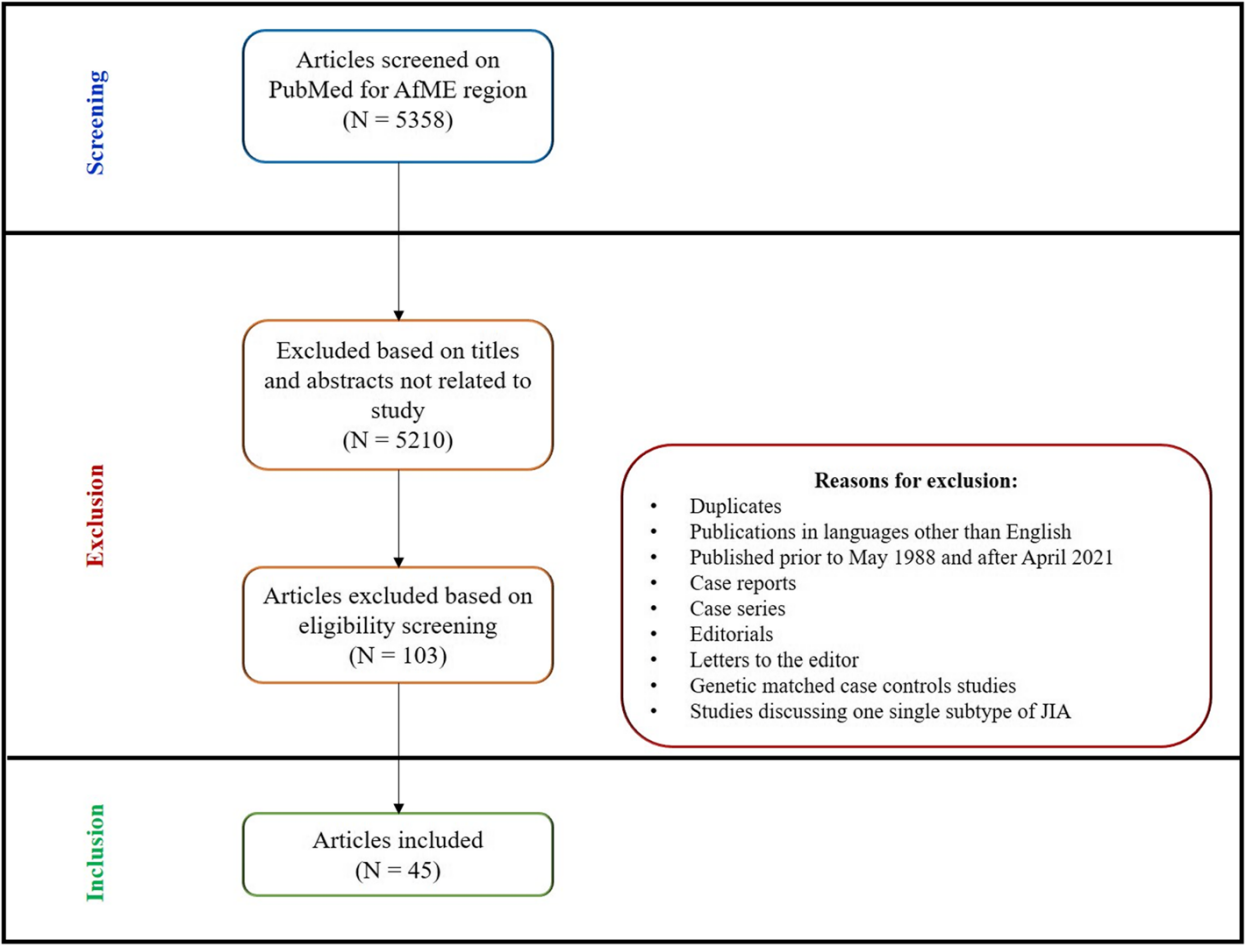
Fig. 1

Publications in languages other than English, evaluating JIA incidence alone, or characterizing one subtype of JIA and or that were published prior to 1988 were excluded. Case reports and case series, editorials, letters to the editor and duplicates were also excluded. For the demographics search genetic matched case controls studies and studies discussing one single subtype of JIA were also excluded to limit selection bias. Please refer to Fig. 1.

Assessment of the risk of bias each study included in our prevalence search was assessed using the Hoy 2012 [9] tool to address of internal and external validity (Table 3). Each parameter was assessed as either low or high risk of bias. Overall assessment of bias was according to number of “high” risk of bias in the parameters per study: low ≤2, moderate [3, 4], and high ≥5.

**Table 3:** Risk of Bias Assessment of Included Studies Using the Hoy 2012 Tool

Sr. No.	Reference	1.Representation	2.Sampling	3.Random Selection	4.Non-response bias	5.Data Collection	6.Case Definition	7.Reliability Tool	8.Method of data collection	9.Prevalence Period	10.Numerators and Denominators	Summary Assessment
1.	Khuffash et al., 1988 [13]	High	Low	Low	Unclear	Low	High	Low	Low	Low	Low	Moderate
2.	Khuffash et al., 1990 [14]	High	Low	Low	Unclear	Low	High	Low	Low	Low	Low	Moderate
3.	Abdwani et al., 2015 [11]	High	Low	Low	Unclear	Low	Low	Low	Low	Low	Low	Low
4.	Ozen et al., 1998 [15]	Low	Low	Low	Unclear	Low	Low	Unclear	Low	Low	Low	Low
5.	El-Soud et al., 2013 [12]	Low	Low	High	Unclear	Low	Low	Low	Low	Low	Low	Low
6.	Singwe-Ngandeu et al., 2013 [16]	High	Low	Low	Unclear	High	Unclear	Low	Low	Unclear	High	High
7.	Tayel et al., 1999 [17]	Unclear	Low	Low	Low	Low	Low	Low	Low	Unclear	Low	Low

All articles included in our search were assessed for their quality in terms of methodology, sample size, study design, classification criteria, study period, characteristics and limitations summarized in (Table 4) and (Table 5) to address wide heterogenicity of design of the study types included and limit potential bias with assessment of the results.

**Table 4:** Characteristics of Studies - Epidemiology

Sr. No.	Reference	Country	Study Design	No. of studies included	Sample size	Single or multiple center	Classification Criteria	Time Period	Study features and Limitations
**Global/Regional**									
1.	Usenbo et al., 2015 [10]	Africa	Systematic Review	27 cross-sectional studies	NA	NA	Multiple criteria	1975-2014	•The studies included do not follow a standardized diagnostic criterion •Risk of bias assessed for each study included •Studies on JIA were not pooled in a meta-analysis due to wide statistical heterogeneity
**Country**									
2.	Khuffash et al., 1988 [13]	Kuwait	Hospital, consultations	NA	186,363	Not reported	ACR (for 3 months)	1978-1987	•10-year study period •ACR criteria utilized •Potential referral bias of more severe cases specifically systemic JIA
3.	Khuffash et al., 1990 [14]	Kuwait	Hospital, medical records revised by experts, hospital attendance	NA	577,540	Multi-center	ACR (for 3 months)	1981-1988	•Retrospective •Large population cohort •Possible underestimation of undiagnosed cases in the community and nonreferral by primary care practitioners •Children aged between 12 and 16 years were excluded. •Female children possibly underrepresented •No current data is available
4.	Abdwani et al., 2015 [11]	Oman	Hospital based, medical records	NA	528,480	Multi-center	ILAR 2004	2004-2013	•Retrospective •10-year study duration •Potential underestimation, only children <13 years of age were included •Potential referral bias, study might have missed on milder cases
5.	Ozen et al., 1998 [15]	Turkey	Community based survey (parent questionnaire, clinical exam in homes by trained practitioners)	NA	46,813	Multi-center	EULAR (for 6 weeks)	1997	•Community-based study from 5 districts in turkey •Possible Exclusion of undiagnosed cases not identifiable from questionnaires may have led to possible underestimation
6.	El-Soud et al., 2013 [12]	Egypt Sharkia Governate, Egypt	Population based prospective study, with retrospective chart review	NA	3,844,718	Multi-center	2004 ILAR	2009-2010	•First population-based study from Sharkia governate •Large population cohort included 19 districts •Possible underestimation of numbers due to undiagnosed cases in the community and nonreferral from primary care practitioners
7.	Singwe-Ngandeu et al., 2013 [16]	Cameroon	Cross sectional medical chart review	NA	34,782	Multi-center	Not reported	2004-2012	•Retrospective •Large population cohort •Potential referral bias of more severe cases
8.	Tayel et al., 1999 [17]	Egypt Alexandria	Community based confirmed by clinical examination	NA	1500	NA	EULAR	NA	•Cross sectional •School based •The prevalence period, method of data collection studied is unclear

**Table 5:** Quality Assessment of Articles Selected – Demographics Results

Sr. No.	Reference	Country	Study Design	N (no. of cases)	Classification Criteria	Time Period	Limitations
**Regional**							
1	Consolaro et al., 2019 [22]	Africa and Middle East	Retrospective chart review with prospective cross-sectional questionnaire	1209	ILAR	2011-2016	•There was disproportionate number of patients included from various geographical areas •Potential underrepresentation of milder forms of JIA and referral bias •Wide variation in tests and evaluation can affect evaluations or tests •Some countries could not be included •Method of grouping some countries in a particular geographical area was arbitrary •Wide variation in healthcare resources across countries
2	Al-Mayouf et al., 2021 [28]	Arab (Saudi Arabia, Libya, United Arab Emirates, Jordan, Oman, Egypt, Kuwait)	Retrospective chart review with prospective disease activity and disease assessment	702	ILAR	2010-2019	•It was a cross-sectional analysis •There is a possibility of patients •selection bias as the participating centers did not enroll the same number of patients •Wide variation in healthcare resources across countries
**Country**							
3	Khuffash et al., 1988 [13]	Kuwait	Hospital, consultations	41	ARA	1978-1987	•10-year study period •ACR criteria utilized •Potential referral bias of more severe cases specifically systemic JIA
4	Khuffash et al., 1990 [14]	Kuwait	Hospital, medical records revised by experts, hospital attendance	108	ARA	1981-1988	•Retrospective •Large population cohort •Possible underestimation of undiagnosed cases in the community and nonreferral by primary care practitioners •Children aged between 12 and 16 years were excluded. •Female children possibly underrepresented •No current data is available
5	Abdwani et al., 2015 [11]	Oman	Retrospective, Hospital, medical records, multicentre	107	ILAR	2004-2013	•Retrospective •10-year study duration •Potential underestimation, only children <13 years of age were included •Potential referral bias, study might have missed on milder cases
6	Ozen et al., 1998 [15]	Turkey	Community based survey (parent questionnaire, clinical exam in homes)	30	EULAR	1997	•Community-based study from 5 districts in turkey •Possible Exclusion of undiagnosed cases not identifiable from questionnaires may have led to possible underestimation
7	Abou El-Soud et al., 2013 [12]	Egypt	Population based in Sharkia Governate prospective study, with retrospective chart review	132	ILAR	2009-2010	•First population-based study from Sharkia governate •Large population cohort included 19 districts •Possible underestimation of numbers due to undiagnosed cases in the community and nonreferral from primary care practitioners
8	Furia et al., 2020 [47]	Tanzania	Retrospective hospital chart review	28	EULAR	2012-2019	•Single centered study •Retrospective study •Possible referral bias and underestimation of milder forms of disease
9	Aiche et al., 2018 [31]	Algeria	Cross sectional survey parent/PRO	70	ILAR	2012-2013	•The objective of the study was to cross-culturally adapt and validate child/adult version of the Juvenile Arthritis Multidimensional Assessment Report (JAMAR) in JIA patients •Possible selection bias •Only selected centers were invited to participate
10	Al Marri et al., 2017 [32]	Saudi Arabia	Prospective record review	23	ILAR	1990-2015	•Potential referral bias could have caused the overall frequency of familial JIA and recurrence risk •Heterogeneous patients were included and were not compared with controls
11	Al-Mayouf et al., 2018 [35]	Saudi Arabia	Cross sectional survey parent/PRO	100	ILAR	2012-2016	•The objective of the study was to cross-culturally adapt and validate child/adult version of the Juvenile Arthritis Multidimensional Assessment Report (JAMAR) in JIA patients Possible selection bias •Only selected centers were invited to participate
12	Salah et al., 2009 [63]	Egypt	Retrospective hospital chart review	196	ILAR	1990-2006	•Single center tertiary hospital study •Higher frequency of oligoarticular JRA, polyarticular and systemic onset JRA could be due to referral bias to tertiary care facilities
13	Al-Abrawi et al., 2018 [33]	Oman	Cross sectional survey parent/PRO	57	ILAR	2012-2013	•The objective of the study was to cross-culturally adapt and validate child/adult version of the Juvenile Arthritis Multidimensional Assessment Report (JAMAR) in JIA patients •Possible selection bias •Only selected centers were invited to participate
14	Demirkaya et al., 2018 [45]	Turkey	Cross sectional survey parent/PRO	466	ILAR	2012-2014	•The objective of the study was to cross-culturally adapt and validate child/adult version of the Juvenile Arthritis Multidimensional Assessment Report (JAMAR) in JIA patients •Possible selection bias •Only selected centers were invited to participate
15	El Miedany et al., 2018 [46]	Egypt	Cross sectional survey parent/PRO	100	ILAR	2014-2015	•The objective of the study was to cross-culturally adapt and validate child/adult version of the Juvenile Arthritis Multidimensional Assessment Report (JAMAR) in JIA patients •Possible selection bias •Only selected centers were invited to participate
16	Hashad et al., 2018 [48]	Libya	Cross sectional survey parent/PRO	100	ILAR	2014-2015	•The objective of the study was to cross-culturally adapt and validate child/adult version of the Juvenile Arthritis Multidimensional Assessment Report (JAMAR) in JIA patients •Possible selection bias •Only selected centers were invited to participate
17	Oyoo et al., 2016 [55]	Kenya	Retrospective hospital chart review	68	ILAR	2009-2016	•Single center tertiary hospital study •Center covers patients from all over Kenya, greater East and Central African region •RF positive polyarthritis patients may be overrepresented which were classified using only one positive assay •Possible underrepresentation of RF negative polyarthritis •Potential referral bias of severe forms of the disease
18	Scott et al., 2018 [57]	South Africa	Cross sectional survey parent/PRO	91	ILAR	2013-2016	•The objective of the study was to cross-culturally adapt and validate child/adult version of the Juvenile Arthritis Multidimensional Assessment Report (JAMAR) in JIA patients •Possible selection bias •Only selected centers were invited to participate
19	Sen et al., 2015 [58]	Turkey	Retrospective hospital chart review	213	ILAR	1998-2013	•Single center study •The collected data may be incomplete and incorrect due to the retrospective study design •HLA-B27 test was not done for all patients
20	Shafaie et al., 2018 [59]	Iran	Cross sectional survey parent/PRO	102	ILAR	2012	•The objective of the study was to cross-culturally adapt and validate child/adult version of the Juvenile Arthritis Multidimensional Assessment Report (JAMAR) in JIA patients •Possible selection bias •Only selected centers were invited to participate
21	Yener et al., 2020 [61]	Turkey	Retrospective hospital chart review	116	ILAR	2012-2018	•Single center study •Retrospective cohort study •The study included lower number of patients as compared to other studies conducted in the country
22	Çakan et al., 2017 [41]	Turkey	Retrospective hospital chart review	265	ILAR	2010-2016	•Single center study •The study included lower number of patients •Short follow-up time
23	Kasapçopur et al., 2004 [51]	Turkey	Retrospective hospital chart review	198	ILAR	NA	•Single center study •Study conducted to determine frequency of ANA positivity and uveitis in newly diagnosed JIA patients
24	Ozdogan et al., 1991 [56]	Turkey	Retrospective hospital chart review	147	EULAR/WHO	1980-1988	•Single center study •Potential referral bias of milder forms of comorbidities such as uveitis
25	Abdul-Sattar et al., 2014 [30]	Egypt	Cross sectional Medical chart review, school attendance records, HRQOL questionnaire	52	ILAR	2011-2013	•Single center study •Included patients aged 7-17 years diagnosed to ILAR criteria •Study aimed to investigate JIA patients school absenteeism and school functioning •Potential selection and referral bias •Cross-sectional study design limits the ability to determine temporal relationships between risk factors and both of school absenteeism and of poor school functioning
26	Abdul-Sattar et al., 2014 [29]	Egypt	Medical chart review, Health related quality of life (HRQoL) questionnaire	58	ILAR	2010-2012	•Single center study •Included patients aged 8-18 years diagnosed to ILAR criteria •Small study sample •Study aimed to identify determinants of impaired HRQOL in children with JIA •Cross-sectional design limits the ability to determine temporal relationships between risk factors and HRQOL
27	Albokhari et al., 2019 [36]	Saudi Arabia	cross sectional health related quality of life survey	44	ILAR	2017	•Single center study •Study aimed to evaluate effect of JIA on HRQOL •Single center study •Potential referral bias and over representation of more severe forms
28	Al-Hemairi et al., 2015 [34]	Saudi Arabia	Retrospective hospital chart review	82	ILAR	2007-2015	•Retrospective record-based study •Single centered •Small sample size •Diagnosis was confirmed by pediatric rheumatologist
29	Amine et al., 2009 [38]	Morocco	Health related quality of life (HRQoL) survey	80	ILAR	2006-2007	•The aim of the study was to assess HRQoL- related impact of JIA •Demographics, subtype, clinical and lab parameters were obtained for patients •Potential selection and referral bias over-representation of severe forms
30	Bahabari et al., 1997 [39]	Saudi Arabia	Retrospective hospital chart review with prospective follow-up	115	ACR	1978-1993	•Multi-center study •18 months follow up •Potential referral bias and under representation of milder forms
31	Bouaddi et al., 2013 [40]	Morocco	Cross-sectional prospective	33	ILAR	2013	•Aim of the study was to assess the impact of JIA on schooling •Single center •Case control •Small sample size
32	Chipeta et al., 2013 [42]	Zambia	Retrospective hospital chart review	78	EULAR/ILAR	1994-1998 and 2006-2010	•Single center •Potential referral bias •Two different classifications were used for each study period •1994-1998 EULAR •2006-2010 ILAR •ANA test was not routinely available
33	Hussein et al., 2018 [49]	Egypt	Retrospective hospital chart review with prospective follow-up	63	ILAR	2004-2010	•Single center •Cross sectional design
34	Olaosebikan et al., 2017 [54]	Nigeria	Retrospective hospital chart review	28	not specified	2010-2016	•Single center •Patients referred to adults rheumatologists due to lack of pediatric rheumatology service •The study included all types of pediatric rheumatology patients, hence unreliable representation of JIA demographics
35	Weakley et al., 2012 [60]	South Africa	Prospective cross sectional	78	ILAR	2010-2011	•Small sample size •Sample bias •Mutli-center
36	Mostafa et al., 2019 [53]	Egypt	Cross sectional HRQol and functional disability questionnaire	48	ILAR	2018	•Aim of the study was to assess functional disability in JIA patients •Single-centered •Potential referral bias and underrepresentation of milder forms
37	Dagher et al., 2014 [43]	Lebanon	Retrospective chart review	66	ILAR	2010-2014	•Single center •Potential referral bias
38	Khawaja et al., 2017 [52]	UAE	Retrospective hospital chart review ICD codes	66	ILAR	2011-2014	•Aim of the study was to assess access to care for JIA patients amongst local and non-local population •Potential referral bias •Selection bias
39	Alzyoud et al., 2020 [37]	Jordan	Retrospective hospital chart review	210	ILAR	2015-2019	•Single center •Potential referral bias •Patients above 14 years of age were not included
40	Demirkaya et al., 2011 [44]	Turkey	Retrospective cross sectional from registry	634	ILAR	2008-2009	•Multi-center •Registry is not representative of all centers from Turkey
41	Karadag et al., 2020 [50]	Turkey	Retrospective hospital chart review with prospective data collection	281	ILAR	2018-2019	•Retrospective chart review •1-year study duration, some patients did not have final diagnosis confirmed •Single center •Potential referral bias
42	Yilmaz et al., 2008 [62]	Turkey	Retrospective chart review	196	ILAR	1995-2004	•Hospital based •Single center •Referral bias may explain low prevalence of oligoarticular JIA and low uveitis

### Search results: epidemiology of JIA in Africa and Middle East

Our PubMed search on epidemiology identified a total of 8 journal publications for all JIA subtypes. The results included 1 systematic review and meta-analysis conducted in Africa between 1975 up to 2014 [10] and seven publications from individual countries [11–17]. One article was excluded from our search as it included only one confirmed JIA case [18].

### Discussion: epidemiology

The prevalence of JIA in Africa and Middle east was noted to be towards the lower range of the global estimate, estimated as (3.8 to 400 per 100,000) [8]. We identified the lowest prevalence in Africa with prevalence rate of less than 3.43 per 100,000, [12, 16] and less than 22 per 100,000 in the Gulf, [11, 13, 14] and highest prevalence identified in Turkey i.e., 64 per 100,000 [15].

Our search identified two studies from Kuwait, [13, 14] that used American College of Rheumatology (ACR) criteria of classification [13, 14] in hospital-based surveys and included patients aged <12 years. The ACR 1978 defined Juvenile Rheumatoid Arthritis (JRA) as persistent arthritis in one or more joints for at least 3 months with exclusion of diseases with similar manifestations. The arthritis was considered polyarticular if five or more joints are involved within 6 months of the onset [19]. The 1988 study extended over a 10-year period (1978-1987) and estimated a prevalence rate of 22 per 100,000 [13]. The other study estimated a prevalence of 18.7 per 100,000 (15.3-22.6) and an incidence of 2.8 (2.3-3.4) per 100,000 [95% CI] [14].

One community based epidemiological study from Turkey, screened 46,813 children from 5 different geographical regions, and reported a prevalence of 64 per 100,000 (43-91 [95% CI]) for juvenile chronic arthritis (including spondylarthritis or psoriatic arthritis) [15]. The EULAR criteria was used which defined Juvenile Chronic Arthritis as the chronic arthritis marked by swelling or effusion, or presence of 2 or more of the following: limitation of range of motion, tenderness or pain on motion, and increased heat in one or more joints for at least 6 weeks and included similar onset types such as juvenile Ankylosing Spondylitis and juvenile Psoriatic Arthritis [20].

Abdwani et al, 2015 conducted a multi-center, medical chart review in Oman between 2004 to 2013, using ILAR 2004 criteria in patients aged <13 years. The prevalence was estimated to be 20 per 100,000 and incidence was reported to be 2 per 100,000 [11].

One Egyptian study screened children <15 years of age in a population based epidemiological study in Sharkia Governate (2009-2010), using the 2004 revised ILAR classification. The prevalence was reported to be 3.43 per 100,000 (3.1–4.3) [95% CI] with overall mean age at diagnosis being 10.5 ± 3.6 (range 4–15) years. There was a statistically noticeable difference between urban and rural populations [12]. Another Egyptian community-based study used The European League Against Rheumatism (EULAR) criteria to confirm and classify cases of Juvenile Chronic Arthritis (JCA) in children aged 10-15 years old. A prevalence rate of 3.3 per 100,000 cases [4–62] [95% CI] was reported [10, 17].

Drawing conclusions on the prevalence of JIA in Africa and Middle East should be approached with caution for several reasons. First, due to the limited number of updated prospective epidemiological studies conducted in the region, and second to the wide heterogeneity of different study designs, case ascertainment and variable study qualities that assessed JIA prevalence in the region.

A wide variance of the prevalence rates was also observed. This variance can be explained by the wide diversity of the healthcare systems capabilities across the region, genetic, disease awareness, smaller sample size, and diagnostic challenges that are more prominent in some countries than others. The variance can also be attributed to absence of electronic healthcare system in some countries, difference in methodologies of case ascertainment, and lack of data collection through registries enough to publish findings. The authors provided Table 4 to outline the quality assessment of articles included from the search and Table 3 to assess the risk of bias for each study included from the search

Our search identified studies with different study designs. Community-based surveys were used in Turkey [15] and Egypt [12] while hospital-based chart reviews were utilized in Oman, Kuwait and Cameroon [11, 13, 14, 16]. Community based prevalence studies are known to provide higher prevalence rates compared to hospital-based studies and allow for undiagnosed cases to be included [8, 21]. Five of the seven local country studies were multi-centered [11, 12, 14–16], and two studies didn’t report details [13, 17]. Only one study conducted in Turkey used diagnostic and clinical examinations to confirm cases [15].

Ideally, studies estimating prevalence should use standardized methods and diagnostic criteria [21] for ascertaining the subtypes from the community and include well trained clinicians experienced in the field of rheumatology to confirm diagnosis. Three of the included studies were conducted more than 24 years ago where study methods, JIA disease and study reporting guidelines have drastically changed and developed. Recent studies tend to better describe the methodology and the results clearly due to evolution of reporting guidelines which was not the case with older studies [21].

JIA nomenclature has changed over the years from JRA to JCA to most recently adopting JIA (Juvenile Idiopathic Arthritis). Over the years, different JIA subtype classifications have been proposed and revisions have been implemented. Hence, the data found with use of a certain classification may reflect changes due to time rather than a real difference because of the classification itself [21, 22].

The variation in results may be attributed to the different classifications (ACR, [13, 14] ILAR, [11, 12] and EULAR [15, 17] used and, in some cases not defining the exact classification used [16].

Variability in disease presentation among the subtypes of JIA may make it difficult to compare prevalence estimates for this condition across different study settings. And like other inflammatory arthritis diseases, extended remissions occur, so that prevalence estimates may include individuals who are experiencing symptoms while cases that are in remission may be missed. Less severe subtypes and symptoms like oligoarticular are not further referred for diagnosis by a specialist pediatric rheumatologist. Most of the country specific prevalence studies set the upper age limit of 12 and 15 years for inclusion [11–14, 17] which can lead to underreporting of patients with onset of symptoms during adolescents between 12-16 years of age [21].

A lack of adequate number of rheumatologists and pediatric rheumatologists further adds to the challenge of accurately estimating the incidence and prevalence of rheumatological diseases [23]. This may contribute to the skewness of the results toward higher prevalence in urban areas.

There are too few pediatricians across the Africa and Middle East region to adequately cater to the JIA population in the region, also an appropriate referral hierarchy would be required to address the gap [24]. Paucity of well-trained pediatric rheumatologists, specifically in the rural areas compel many patients to visit other traditional healers [25] or healthcare professionals like general practitioners, family physicians [24] or orthopedics rather than rheumatologists.

Awareness of JIA is increasing and is reflected in the increasing prevalence across the globe and the region [26]. As healthcare systems and economies are developing, more resources are allocated towards improving diagnosis and management of childhood illnesses. Noticeably, most data in the literature describes evidence from the Middle East and North Africa region. There are far fewer data available on prevalence from the sub-Saharan Africa region. The absence of data, however, does not imply absence of the disease.

Robust epidemiological data is needed from the region to assess the impact of JIA on children from Africa and the Middle East through the development of prospective community based epidemiological studies covering regions rather than individual country-based studies needed to accurately determine the prevalence of JIA across the region. In addition, the development of national and regional registries can further facilitate the generation of evidence on JIA prevalence from this region [9].

Other solutions include increased capacity of general health care practitioners and pediatric rheumatologists to address healthcare access for patients underdiagnosed or undertreated. In addition, raise awareness to general and specialized practitioners on MSK examination skills and define uniform case ascertainment or referral criteria [27].

### Search Results: Demographics

Our literature search identified 42 articles describing JIA subtypes and demographics from Africa and Middle East. We identified one global study that included 1209 patients from Africa and Middle East, [22] and one multicenter regional study from seven Arab countries, [28] and 40 publications of data from individual countries [11–15, 29–63]. A summary of the demographics is presented in Table 2**.**

### Discussion: Demographics

The findings of this review support that the most prevalent subtype in Africa and Middle East is oligoarticular JIA subtype, followed by polyarticular RF negative, and systemic subtype. Our findings support the global epidemiology, treatment, and outcome of childhood arthritis throughout the world (EPOCA) study findings [22] and the regional Pediatric Rheumatology Arab Group (PRAG) study [28].

Oligoarticular subtype was observed to be the most frequent subtype based on the 15 local studies [12, 15, 29–31, 37, 38, 43, 44, 49, 50, 57, 59, 62, 63]. Followed by polyarticular then systemic JIA.

On a regional scale, the EPOCA study, enrolled 1209 JIA patients using ILAR 2004 criteria, from 15 participating countries from Africa and Middle East region. The study identified oligoarticular JIA (37.8%), RF-negative polyarthritis (22.4%) and systemic JIA (16.9%) as the predominant subtypes in Africa and the Middle East. A predominance of the female gender (61.6%) was observed with mean age of onset of 6.0 (2.9-9.8) and 5.9% of cases had positive signs of uveitis with predominance of uveitis amongst oligoarticular sub-type in 12.4% of the cases from the region [22].

In the PRAG study, 702 JIA patients with a disease duration of more than one year and fulfilled the ILAR criteria were enrolled from 14 pediatric rheumatology centers across seven Arab countries. Oligoarticular JIA (34.9%) was identified as the predominant subtype. Polyarticular JIA (29.5%) and systemic JIA (24.5%) were the second and third most identified subtypes [28].

Oligoarticular subtype has also been the most common across all regions in Europe and North and Latin America except Southeast Asia [8, 22, 64, 65]. A similar finding has also been observed from a JIA epidemiological study conducted in Canada that focused on ethnicity as a risk factor in JIA phenotypes [66]. Arab descent patients had a predominance of oligoarticular subtype [66]. Patients of Arab descent had the highest predominance of systemic disease subtype, almost twice higher than Asian descent patients 23.5% vs. 12%. In contrast, African descent patients had an equal distribution of oligoarticular and RF negative polyarticular disease and had the highest RF positive polyarticular disease prevalence amongst all ethnicities at 16.1% [66].

RF negative polyarticular JIA was the second most identified subtype in Africa and Middle East. The RF negative subtypes were reported to be the predominate subtype in Kuwait, [13, 14] Oman, [11, 33] and Saudi Arabia [35]. One study from Morocco reported predominance of RF-positive polyarthritis [40]. And only one study from Egypt identified undifferentiated subtype (40%) to be predominant [46]. Globally, RF negative polyarticular JIA was recognized to be most prevalent in North America and least in Southeast Asia [22]. Regionally, RF negative polyarticular JIA was identified at 22.6% from the PRAG study, [28] and 22.4% from the EPOCA study [22].

One study from Morocco (45.5%) [40] and one study from Egypt (25.4%) [49] reported a higher prevalence of RF positive polyarthritis as compared to RF negative subtype. The exact cause for a higher frequency of RF positive polyarthritis is unknown but can be attributed to genetics and selection bias. Among the studies that tested and reported rheumatoid factor results, Jordan reported the lowest RF positivity at 3.8% [37]. Regionally, RF positive polyarthritis was identified from the PRAG study at 6.8% [28] and 5% from the EPOCA study [22]. In the Canadian multiethnic cohort study, patients with African descent had the highest prevalence of RF positive polyarthritis and a lower uveitis rate [66]. This observation has been made in multiple studies describing the African population [67, 68]. The subtype frequencies of various geographic regions are presented in Table 6.

**Table 6:** Frequency of ILAR Categories by Geographic Area

	Northern Europe (***n*** = 845)	Western Europe (***n*** = 832)	Southern Europe (***n*** = 2400)	Eastern Europe (***n*** = 2044)	North America (***n*** = 523)	Latin America (***n*** = 849)	Africa and Middle East (***n*** = 1209)	Southeast Asia (***n*** = 379)
**Systemic arthritis**	42 (5.0)	57 (6.9)	204 (8.5)	167 (8.2)	22 (4.2)	149 (17.6)	204 (16.9)	125 (33.0)
**Oligoarticular**	340 (40.2)	317 (38.1)	1360 (56.7)	848 (41.5)	185 (35.4)	261 (30.7)	457 (37.8)	41 (10.8)
**RF-negative polyarthritis**	223 (26.4)	198 (23.8)	480 (20.0)	539 (26.4)	165 (31.5)	217 (25.6)	271 (22.4)	48 (12.7)
**RF-positive polyarthritis**	30 (3.6)	22 (2.6)	31 (1.3)	91 (4.5)	22 (4.2)	95 (11.2)	61 (5.0)	30 (7.9)
**Psoriatic arthritis**	35 (4.1)	40 (4.8)	88 (3.7)	54 (2.6)	37 (7.1)	13 (1.5)	37 (3.1)	5 (1.3)
**Enthesitis related arthritis**	87 (10.3)	125 (15.0)	130 (5.4)	254 (12.4)	56 (10.7)	83 (9.8)	111 (9.2)	113 (29.8)
**Undifferentiated arthritis**	88 (10.4)	73 (8.8)	107 (4.5)	91 (4.5)	36 (6.9)	31 (3.7)	68 (5.6)	17 (4.5)

Data are number (%)

ILAR = International League of Associations for Rheumatology

Reprinted from *Lancet Child Adolesc Health*; 2019 3 (4):255-63. Reproduced with permission from copyright holder.

Notably, most of the Saudi Arabia studies reported systemic JIA subtype to be the most frequent [32, 34–36, 39] and in only one study from Turkey (26.3%) [51]. Saudi Arabia was the only country that reported systemic subtype as the most frequent from multiple studies [32, 35, 36, 39]. Higher incidence of systemic JIA was associated with large familial clusters in the country, especially in the southern region [32, 69]. Familial JIA suggest an autosomal recessive mode of inheritance with specific mutations in genetic markers like LACC1 [70, 71]. It has been observed that familial systemic JIA patients were younger at the onset of disease and diagnosed earlier than sporadic JIA cases and had a predominance of refractory disease with progressive disease course [32]. These findings were attributed to a high consanguinity marriage, and potential referral bias (severe cases presentation) [32, 35, 69]. Systemic JIA was identified at 16.9% from Africa and Middle East region in the EPOCA study [22] and identified at higher prevalence of 24.5% was observed in the PRAG study [28]. A lower frequency of systemic JIA subtype was observed in studies from Turkey [50] and South Africa [57] at 3.9% and 4.4%, respectively.

Enthesitis related arthritis (ERA) subtype was most frequent from three retrospective chart studies from Turkey, reported at 34.5% from Istanbul, [50] 32.9% from Denizli region [41] and 32.8% from the Adana region [61]. A third study from Istanbul identified ERA as the second most frequent subtype in 21.7% of the cases analyzed [51]. The lowest frequency of ERA subtype was reported from Saudi Arabia (1.2%), [34] United Arab Emirates (1.5%) [52]. It was observed that several studies from Iran, [59] Oman, [33] Saudi Arabia, [32, 36, 39] and Egypt [49, 53] reported no ERA cases in their cohort. However, two studies from South Africa (23% and 15.4%) [57, 60] reported higher prevalence of ERA subtypes than others. The trend for the high frequency of ERA in South Africa was attributed to the high population of people of Asian and European descent in some regions in South Africa [60].

EPOCA study identified ERA subtype in 9.2% of all cases in Africa and Middle East region, and PRAG study at 5.6% of all JIA cases [22, 28]. This finding of higher predominance of boys in one Turkish study was attributed by high frequency of ERA in Turkey which is more frequent in males than in females [41].

ERA subtype was identified at 9.2% and 5.6% from the EPOCA and PRAG studies, respectively [28]. And globally, ERA has been highest among southeast Asia and lowest in Southern Europe [22, 66]. The possible reason for the lower prevalence of ERA in the Arab and African populations is unknown but can explained by higher incidence of ERA in post-pubertal male, which may be referred to adult rheumatologists and not counted as JIA in pediatric rheumatology literature. Arab ERA patients showed greater articular damage with significant limitation [28]. Intra-country differences were observed in the frequency of JIA subtypes in Turkey [61]. Denizli and Istanbul regions reported ERA as the most common subtype, [41, 61] while oligoarticular was the most prevalent subtype in Adana, [62] Diyarbakir, [58] and from a regional multi-center registry study in Turkey [44]. The heterogenic nature of the Turkish population, cultural, socioeconomic, food habits, and mixed ethnicities have resulted in region wide variations [50, 61].

Psoriatic arthritis and undifferentiated arthritis were the least reported JIA subtype across all the studies from the region, and this observation is aligned with other regions globally [22].

In various studies conducted across the globe, an overall female predominance for JIA was observed [8, 22]. Our literature review also supports that JIA is more likely to occur in girls than in boys in the region [22]. However, notable differences in the ratios exist across the different countries in the region. We observed a higher female to male ratio in most studies conducted in individual countries from Africa and Middle East [11–13, 29–39, 42, 44, 45, 48, 52, 53, 55, 57–59, 61, 63]. Eight studies reported number of male cases to be higher in comparison to female cases. These included five studies from Turkey (female to male ratio - 0.94:1 [41], 0.6:1 [15], 0.87:1 [51], 0.92:1 [62], and 0.77:1 [56],) two from Egypt (female to male ratio - 0.9:1 [49] and 0.88:1 [46]), and one from Morocco (female to male ratio - 0.83:1 [40]). Notably, studies from Lebanon, Kuwait, South Africa, and Tanzania cohorts showed near equal gender distribution [14, 43, 47, 60]. In various studies conducted across the globe, an overall female predominance for JIA was observed [8, 22]. A similar trend was observed in most studies conducted in individual countries from Africa and Middle East [11–13, 29–39, 42, 44, 45, 48, 52, 53, 55, 57–59, 61, 63]. The multinational EPOCA [22] and PRAG [28] studies identified a predominance of girls in the identified JIA cases. The female to male ratio ranged from 1.6:1 [22] to 2:1 [28].

It is noticeable that there is female predominance in many autoimmune diseases, however, the referral bias and study methodologies, case ascertainment and geography can contribute to the variance in gender ratios [72–74]. Male predominance has been reported in some studies that maybe explained by unequal school and medical care provided to male and female children, especially in the rural areas [14, 21]. Globally two studies identified higher prevalence of disease in girls than in boys 19.4 (18.3-20.6) per 100,000 and 11.9 (10.2-11.9) per 100,000 [95% CI], respectively [8]. The higher predominance of JIA in boys has also been linked to high frequency of ERA by one Turkish study [41].

ANA positivity was identified in 30.9% of cases from the PRAG study [28]. From the local studies, the lowest frequency of ANA was reported in a study from Egypt (0%) [46] and highest from Morocco (76%) [40]. Other studies that reported relatively higher ANA positivity rates included 48.5% from Egypt [12], 44% from Turkey [61] and 36.5% from Saudi Arabia [34]. Notably, several local studies reported no ANA-positive patients in all its cohort. Our findings from this review conclude that a wide heterogeneity in ANA positivity among JIA studies can be attributed to genetics, different methods of ANA ascertainment and the unavoidable referral bias.

The human leukocyte antigen (HLA) - B27 was identified regionally in 5.3% cases by the PRAG study [28]. The majority of studies did not test for HLA-B27 in all patients, and some opted to test HLA-B27 in suspected ERA cases only. Among those studies, an Egyptian study reported 66% positivity, a South African study reported 23% positivity, and a Turkish study reported 63.3% positivity in the confirmed ERA cases [12, 44, 60]. One study from Turkey tested HLA-B27 in all ERA phenotype cases and in males over six years of age and reported 26% positivity rate [41]. One study analyzed HLA-B27 in all its patients [39]. One of the studies that analyzed HLA-B27, all JIA subtypes reported 21.1% positivity in overall cohort. However, all HLA-B27 positive patients were of ERA subtype [61].

Our findings from this review observed that uveitis and ANA positivity rates seem to be low for Africa and Middle East region. In individual countries, uveitis’ prevalence ranged from 1% from Iran [59] to 19.7% from Egypt [12]. Uveitis was identified in 8.3% of the PRAG study cases [28] and 5.9% from the EPOCA study [22]. The EPOCA study observed the lowest prevalence of uveitis in Africa and Middle East as compared to other regions [22] (Refer to Table 7). PRAG study reported a higher rate of uveitis i.e., 8.3% [28]. Two studies from Oman reported zero cases of uveitis from their cohorts [11, 33]. We identified one outlier study from Egypt, that reported 19.7% of the cohort with evidence of uveitis predominantly in the oligoarticular subtype. Coincidently, the same study reported high ANA positivity in its cohort in 48.5% cases and a high frequency of both combined ANA positivity and uveitis in oligoarticular subtype 62.3% [12]. Saurenman *et al*, 2007 also reported a lower relative risk of developing uveitis in Arab and Asian descent patients than European or native North American ethnic groups [66]. Similar findings have been observed in the African population [67, 68].

**Table 7:** Demographic Features and Frequency of Uveitis

	Northern Europe (***n*** = 845)	Western Europe (***n*** = 832)	Southern Europe (***n*** = 2400)	Eastern Europe (***n*** = 2044)	North America (***n*** = 523)	Latin America (***n*** = 849)	Africa and Middle East (***n*** = 1209)	Southeast Asia (***n*** = 379)
**Girls**	593 (70.2%)	538 (64.7%)	1763 (73.5%)	1303 (63.7%)	374 (71.5%)	550 (64.8%)	745 (61.6%)	164 (43.3%)
**Boys**	252 (29.8%)	294 (35.3%)	637 (26.5%)	741 (36.3%)	149 (28.5%)	299 (35.2%)	463 (38.3%)	215 (56.7%)
**Age at onset (years)**	4.7 (2.2 – 9.4)	6.4 (2.7 – 10.4)	3.5 (1.9 – 7.3)	6.7 (3.0 – 10.7)	7.4 (3.1 – 10.9)	6.8 (3.6 – 10.5)	6.0 (2.9 – 9.8)	7.0 (3.9 – 10.7)
**Interval onset-referral (years)**	0.3 (0.1 – 0.8)	0.4 (0.2 – 1.0)	0.3 (0.1 – 0.9)	0.3 (0.1 – 1.0)	0.3 (0.1 – 0.8)	0.4 (0.2 – 1.0)	0.4 (0.2 – 1.5)	0.6 (0.2 – 2.0)
**Disease duration (years)**	5.0 (2.5 – 8.4)	3.8 (1.8 – 6.7)	4.4 (1.9 – 7.7)	3.4 (1.6 – 6.2)	4.4 (1.9 – 8.0)	4.6 (2.1 – 7.3)	2.8 (1.2 – 5.4)	3.9 (1.9 – 6.7)
**Uveitis**	161 (19.1%)	94 (11.3%)	450 (18.8%)	183 (9.0%)	59 (11.3%)	54 (6.4%)	71 (5.9%)	19 (5.0%)

Data are n(%) or median (IQR)

Reprinted from *Lancet Child Adolesc Health*; 2019 3 (4) :255-63. Reproduced with permission from copyright holder.

Across many studies conducted on JIA subtypes worldwide, a wide heterogeneity in the pattern of disease, age of onset, sex, and phenotypes has been observed [22, 66] owing to factors such as immunogenetic, socioeconomic status, environment, and diagnostic criteria [21, 61]. The wide diversity of study design and diagnostic criteria used adds to the challenge of forming a reliable picture of the demographics in the region. Further, there is a lack of uniformity with regards to the type and definition of biomarkers tested (RF, HLA-B27, ANA) and the subtype they are tested in [21, 66]. In some countries, there could be a recruitment bias in studies for patients >10 years of age, as they consult an adult rheumatologist [40]. Factors that may influence the heterogeneity in JIA subtype frequency within the region included: diverse socioeconomic, cultural, nutritional habits and genetics. Migration between the different parts of the region results in mixed ethnicities and different genetic constructs and could significantly contributor to this heterogeneity [66].

The readers should note that the observations should be approached with caution owing to the heterogenicity of the studies pooled. Most of the studies included in this manuscript for reviewing the demographics are single-centered, retrospective study with notable selection biases. Some of the studies included were limited by their sample size.

### Region-specific unmet needs

Several factors can contribute to the delays in proper diagnosis and management of JIA which vary region wise. The challenges include access to rheumatology services, access to proper diagnosis and therapies, and lack of awareness of rheumatic musculoskeletal disorders at the policymaker and public level and general pediatricians [23, 24]. Limited access to rheumatologists has been identified as a global challenge, which has also been reported in Africa than in Middle East region. The ratio of practicing rheumatologists ranged 0.3-0.89 rheumatologists per 100,000 in the Gulf and reported lower in Africa 0-0.01 per 100,000 compared to 1.78 per 100,000 in USA [23]. This challenge is further amplified for pediatric patients due to the even greater limitation of pediatric rheumatologists' access and pediatric rheumatology training [24, 75]. The disparities in regulatory approval timelines, health care system settings, economies, and the level of a financial burden on patients may vary considerably across Africa and Middle East.

International guidelines recommend initiating treatment soon after diagnosis and setting remission of disease as the optimal treatment target [76–78]. Those with a longer duration of un-or undertreated disease may only achieve minimal improvement in disease activity. There are limited local and regional guidelines, International guidelines exist but are not always applicable in the region because of the high costs of new therapies and the constraints of regular follow-up. Algeria has developed their national JIA treatment guideline and is published in French [79]. In Egypt, registries have been set up to advance the cause and local guideline is underdevelopment.

A recommendation for management of JIA in less resourced countries has also been developed in a global effort which included experts from South Africa, Kenya and Zambia [80]. At the same time, other countries follow established international guidelines such as ACR, EULAR [35, 76–78]. There are regional collaborations being established throughout the region between countries under PRAG group which is a part of the Arab League of Associations for Rheumatology (ArLAR). The aim of these collaborations is to develop the field of pediatric rheumatology in the region, provide a network of research collaboration to address the unmet needs for patients, develop a consensus on JIA evidence generation and local treatment guidelines. As stated by an ongoing Pediatric Task Force Global Musculoskeletal Health there is a real need to improve research and outcomes for musculoskeletal disorders [81]. There are initiatives like Pediatric Society of the African League Against Rheumatism (PAFLAR) and Global Task Force for Musculoskeletal Health and Pediatric Rheumatology European Society (PReS), who have recognized the need and are working towards reaching out to children with rheumatic diseases who do not have access to proper care [82].

## Conclusion

The region of Africa and Middle East is very diverse in terms of socioeconomic conditions, environmental factors, ethnicities, and healthcare infrastructures. There is a paucity of the latest and adequate data on JIA on its epidemiology. In the absence of databases or registries to track disease progression, JIA data for Africa and Middle East are generally derived from hospital-based studies, providing limited accounts of epidemiology. Prospective, population-based studies are preferable in descriptive epidemiology, compared to studies using secondary data that depend upon hospital or public health registry systems. However, such studies are expensive, time-consuming, and consequently rare, especially in lower-income countries. Hence, a comprehensive review was planned to critically analyze the available data from the region. The prevalence rates of the region are relatively lower compared to the global estimates. The reasons for the wide range reported from the region include differences in study designs, methodologies, reach to healthcare facilities, and non-uniform study methodologies. From the demographic data gathered, it was concluded that the oligoarticular subtype was the predominant one over another subtype in Africa and Middle East. It was also noted that the incidence of uveitis and ANA positivity in Africa and Middle East region was lower as compared to the incidence from other parts of the world. The region has an evident unmet need for awareness, delayed diagnosis, lack of an adequate number of rheumatologists, no published local or regional guidelines, and economic disparities. These lacunae need to be addressed to effectively manage JIA in the region.

## Data Availability

Not applicable

## Abbreviations

ACR: American College of Rheumatology
ANA: Anti-nuclear antibody
ArLAR: Arab League of Associations for Rheumatology
EPOCA: The multinational epidemiology, treatment, and outcome of childhood arthritis throughout the world
ERA: Enthesitis related arthritis
EULAR: The European League Against Rheumatism
HLA: Human leukocyte antigen
ILAR: International League of Associations for Rheumatology
JCA: Juvenile Chronic Arthritis
JIA: Juvenile Idiopathic Arthritis
JRA: Juvenile Rheumatoid Arthritis
PAFLAR: Pediatric Society of the African League Against Rheumatism
PRAG: Pediatric Rheumatology Arab Group
PReS: Pediatric Rheumatology European Society
RF: Rheumatoid Factor
